# Motion Cancellation Technique of Vital Signal Detectors Based on Continuous-Wave Radar Technology

**DOI:** 10.3390/s25072156

**Published:** 2025-03-28

**Authors:** Min-Seok Kwon, Yuna Park, Joo-Eun Park, Geon-Haeng Lee, Sang-Hoon Jeon, Jae-Hyun Lee, Joon-Hyuk Yoon, Jong-Ryul Yang

**Affiliations:** Department of Electrical and Electronics Engineering, Konkuk University, Seoul 05029, Republic of Korea; suk4215@konkuk.ac.kr (M.-S.K.); engle0108@konkuk.ac.kr (Y.P.); apriljepark@konkuk.ac.kr (J.-E.P.); lgh0908@konkuk.ac.kr (G.-H.L.); pano511@konkuk.ac.kr (S.-H.J.); reenact11@konkuk.ac.kr (J.-H.L.); yoonkevin@konkuk.ac.kr (J.-H.Y.)

**Keywords:** continuous wave, Doppler radar, modulated signals, motion cancellation, radar sensors, random body motion, vital sign detectors

## Abstract

Continuous-wave (CW) radar sensors can remotely measure respiration and heartbeat by detecting the periodic movements of internal organs. However, external disturbances, such as random body motion (RBM) or environmental interference, significantly degrade the signal-to-noise ratio (SNR) and reduce the accuracy of vital sign detection. The various motion cancellation techniques that have been proposed to enhance robustness against RBMs include improvements in radar architecture, advanced signal processing algorithms, and studies on electromagnetic propagation characteristics. This paper provides a comprehensive review of recent advancements in motion cancellation techniques for CW radar-based vital sign detectors and discusses future research directions to improve detection performance in dynamic environments.

## 1. Introduction

The demand for non-contact health monitoring systems has grown significantly in recent years, driven by applications in healthcare, elderly care, stress management, and sleep studies. Among the various sensing modalities available, continuous-wave (CW) radar sensors have emerged as a compelling choice because they can detect vital signs, such as respiration and heartbeat, without direct physical contact. Several CW-based radar technologies, specifically CW, frequency-modulated CW (FMCW), and pulse-modulated CW (PMCW), offer distinct advantages that make them well suited for non-contact vital sign detection. For instance, CW radar provides a simple and cost-effective implementation that exhibits high sensitivity to subtle movements. FMCW radar achieves enhanced range resolution, enabling the precise differentiation of multiple subjects or diverse types of motion, and PMCW radar further improves noise robustness in cluttered environments [1]. These strengths, combined with ongoing advancements in signal processing and hardware design, have led to the widespread adoption of CW-based radar in non-contact health monitoring applications.

Despite these advantages, CW radar-based vital sign detection still faces several critical challenges. Motion artifacts caused by random body motion (RBM) or nearby objects pose significant obstacles to accurate measurements, with environmental factors such as multipath interference and clutter further degrading signal quality. Among these issues, RBM, which encompasses involuntary or unpredictable subject movements unrelated to targeted physiological signals, has the most substantial impact on detection accuracy. These movements introduce considerable noise into the received radar signal, making it difficult to isolate and measure the small displacements associated with respiration and heartbeat. Consequently, RBM can dramatically reduce the signal-to-noise ratio (SNR), induce phase distortion, and lead to imprecise extraction of vital sign information. The degradation in radar performance not only decreases measurement reliability but also diminishes the effectiveness of CW radar-based monitoring systems.

To address these challenges, numerous motion cancellation techniques have been proposed to suppress the effects of RBM and enhance the robustness of CW radar-based vital sign detection. Both involuntary and voluntary movements, such as limb motions, posture shifts, and external disturbances, introduce substantial errors into the measurement process, making motion cancellation a pivotal aspect of system design. Recent approaches have leveraged advanced signal processing algorithms, machine learning models, and hardware innovations to differentiate physiological signals (e.g., respiration and heartbeat) from unintended motion artifacts.

This paper provides a comprehensive review of state-of-the-art motion cancellation strategies for CW radar-based vital sign detection, focusing on three primary areas:

1. Radar architecture and design modifications;
2. Signal processing algorithms for motion artifact suppression;
3. Studies on propagation characteristics and environmental impact mitigation.

By categorizing existing methods into these topics, this study highlights current trends, identifies key technical obstacles, and discusses future research directions for improving the performance and reliability of CW radar-based sensors for non-contact health monitoring systems.

## 2. Continuous-Wave Radars for Vital Sign Detection

Continuous-wave Doppler radar detects variations in the Doppler frequency of the transmitted CW signal caused by respiratory-induced chest motion and periodic movement of the heart, thereby extracting respiration and heartbeat signals from changes in the transmitted and received wave characteristics [2,3,4]. Accordingly, CW radar has become a pivotal technology for non-contact vital sign detection. In CW radar-based vital sign monitoring, common performance metrics include the SNR relative to RBM noise and measurement accuracy (or error rate) when comparing respiration and heartbeat signals with reference data [2].

In general, human body motion involves significantly larger displacements than those induced by respiration (1–12 mm) or heartbeat (0.2–0.5 mm). Consequently, commercializing non-contact vital sign detection technology using CW Doppler radar in the industrial and medical fields requires mitigating the impact of subject motion. When a person moves, highly sensitive receivers designed for heartbeat detection may become saturated because of limitations in the dynamic range, rendering the system unable to detect the target signals [3]. Moreover, even if the radar possesses a sufficiently wide dynamic range, the frequency components generated by body motion may overlap the frequency bands associated with respiration and heartbeat [4]. These extraneous frequency components act as noise, thereby reducing the SNR in vital sign detection and, in extreme cases, making accurate detection impossible [5].

### 2.1. Principle of CW Radar for Vital Sign Detection

**Figure 1:**
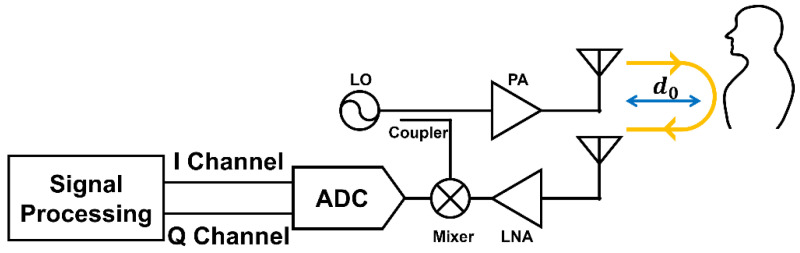
Block diagram of a CW radar system for vital signal detection.

Figure 1 shows a schematic of the CW radar system used for detecting vital signals from a subject located at a distance *d*_0_ from the radar. A CW radar operates by continuously transmitting electromagnetic (EM) waves at a designated frequency and detecting motion via the reflected signal. The slightest body motion (e.g., respiration and heartbeat) causes phase changes and Doppler frequency shifts in the reflected signal, which can be analyzed to extract the vital signs [6].

The transmitted signal of a CW radar can be represented as

(1) $$s_{t}(t)=A_{t}\cos(2\pi \;f_{c}t),$$

where *f*_c_ denotes the transmitted frequency, and *A* denotes the amplitude of the transmitted signal. To consider respiration and heartbeat movements, the signal received from the stationary subject can be modeled to include a time-varying phase *ϕ*(*t*), as follows:

(2) $$s_{r}(t)=A_{r}\cos(2\pi \;f_{c}t+\phi (t)),$$

(3) $$\phi \left(t\right)=4\pi \frac{d_{0}+x\left(t\right)}{\lambda },$$

(4) $$x\left(t\right)=A_{hr}\cos\left(2\pi \;f_{hr}t\right)+A_{rr}\cos(2\pi \;f_{rr}t),$$

where *d*_0_ is the average distance between the target and radar, *x*(*t*) represents the displacement caused by respiration and heartbeat, and *λ* is the wavelength of the transmitted signal. Detailed extraction of *ϕ*(*t*) allows the separation of respiration and heartbeat components.

### 2.2. Challenges in Vital Sign Detection

Non-contact vital sign detection using CW and FMCW radars is hindered by several technical constraints [7,8]. Stationary objects around the body may interfere with the received signal [7]; however, they can be easily removed as they do not change over time. Issues that do not follow a consistent pattern such as body movement artifacts, environmental disturbances, and sensor placement/sensitivity are far more dominant in vital sign detection. These factors significantly influence measurement accuracy and reliability, often increasing detection errors and undermining the performance of radar-based biomedical systems.

#### 2.2.1. Body Motion Artifacts

Since CW and FMCW radar systems extract vital signs from subtle movements of the human body, sudden or voluntary body motions (e.g., arm movements, turning, coughing, or postural changes) can introduce significant phase shifts and Doppler effects that degrade measurement accuracy. Even minor involuntary movements over extended measurement periods can result in the following [2,3,5]:

- Reduced SNR: Large-amplitude motion artifacts overshadow weaker signals from respiration or heartbeat.
- Distorted phase: Rapid or erratic movements affect the received phase information, complicating vital signal extraction.
- Unexpected frequency shifts: Body motion can generate frequency components similar to those of respiration and heartbeat signals, making it challenging to distinguish genuine vital sign data.

Body motions produce signals with larger amplitudes than respiration or heartbeat, which pose the most formidable challenge in radar-based vital sign detection. Consequently, motion cancellation techniques are indispensable for mitigating these artifacts.

#### 2.2.2. Environmental Disturbances

CW and FMCW radar systems are sensitive to a variety of environmental factors that can degrade signal quality [9,10]:

- Multipath interference: Radar signals may reflect off walls, furniture, and other objects, thereby creating multiple propagation paths. These multipath reflections introduce phase and amplitude distortions that hinder accurate signal interpretation, particularly in dense indoor settings.
- Electromagnetic interference (EMI): Radar sensors operate in specific frequency bands; therefore, interference from Wi-Fi, Bluetooth, 5G base stations, or medical equipment can lower the SNR, rendering vital sign detection unstable or unreliable.
- Multi-subject detection: When multiple individuals are present, accurately separating each subject’s vital signals becomes challenging. Techniques such as multiple-input multiple-output (MIMO) radar may be required to isolate individual respiration and heartbeat signals.

#### 2.2.3. Sensor Sensitivity and Placement

The performance of CW and FMCW radar systems additionally depends on sensor sensitivity and placement [4,11], involving the following considerations:

- Importance of optimal placement: Improper sensor placement may lead to reduced SNR and phase detection errors. Direct alignment with the thoracic or cardiac regions is typically recommended for accurate measurements.
- Body posture and signal attenuation: Different postures, such as sitting, standing, or lying down, alter the reflection patterns. Changes in subject-to-sensor distance can further attenuate signals, leading to reduced detection accuracy.
- Weak signal strength: Because respiration and heartbeat movements inherently generate low-amplitude signals, advanced techniques (e.g., signal amplification, high-resolution fast Fourier transform (FFT)) are required to improve system sensitivity.

Among these challenges, RBM remains the most challenging obstacle for CW and FMCW radar-based vital sign detection, making motion cancellation techniques essential. The following sections examine various motion cancellation methods, including radar architecture modifications, signal processing enhancements, and approaches that manage environmental factors. Researchers have aimed to mitigate body motion noise, improve measurement accuracy, and refine system robustness for practical biomedical applications.

## 3. Motion Cancellation Techniques

### 3.1. Radar Architecture Enhancements

Non-contact radar-based vital sign detection has garnered significant attention in applications ranging from medical monitoring to driver status sensing and smart home care. However, the RBM in real-world scenarios introduces signal distortion and degrades detection accuracy. To address this challenge, researchers have explored structural and hardware-level enhancements that improve signal quality and robustness against motion artifacts. This section reviews the key architectural advances in radar systems designed to mitigate RBM while accurately extracting physiological signals.

#### 3.1.1. Multi-Antenna and MIMO Systems

**Figure 2:**
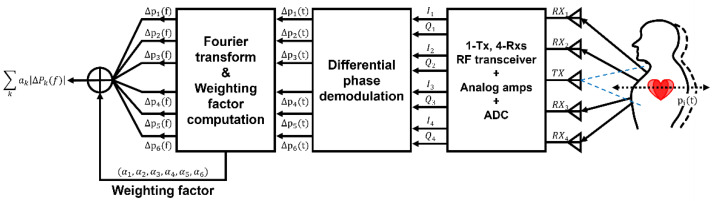
Block diagram of multi antenna radar to reduce body motion artifacts and common noise (reproduced with permission from the corresponding author, Differential phase Doppler radar with collocated multiple receivers for noncontact vital signal detection, published by IEEE, 2019) [12].

Multi-antenna and MIMO radar configurations are crucial for enhancing SNR and improving multi-target detection. In traditional single-antenna systems, signals are transmitted and received via a single path, rendering them vulnerable to motion-induced distortions and target overlap. Thus, recent research has focused on increasing spatial diversity using multi-antenna arrays and employing differential beamforming techniques. Figure 2 presents a block diagram of the multi-antenna radar system used to reduce body motion artifacts and suppress common noise, thereby enhancing detection accuracy.

- A differential phase Doppler radar system using a collocated multiple-receiver array effectively removes common motion components by analyzing the phase differences among the receiver channels [12]. This approach demonstrated an improvement of over 20 dB in noise suppression relative to the single-antenna setup.
- A virtual array-based radar system uses Pearson correlation coefficients to identify highly correlated regions among multiple measurement points on the chest [13]. A Kalman filter tracked these regions to suppress artifacts, achieving heartbeat and respiration detection errors within 5 bpm under dynamic conditions (motion speeds of up to 0.5 m/s).
- Null-point beamforming with a 60 GHz FMCW MIMO radar successfully reduced common body motion artifacts by 18 dB, enabling simultaneous vital sign detection in two individuals [14]. Independent signals are allocated to each target to facilitate motion compensation under random conditions.
- In an LFMCW radar with multiple receivers, a four-receiver array combines multi-channel Kalman smoothing with local hidden Markov models to improve the SNR by up to 7.5 dB [15]. This approach maintained respiration and heartbeat detection errors below 2 bpm in RBM.
- MIMO-based radar has also proven effective in in-vehicle environments, where driver motion and vehicle vibration degrade performance [16]. Digital beamforming effectively isolated the driver’s vital signals from extraneous reflections, yielding a heart rate detection error of 0.82 bpm and a respiration rate error of 0.16 rpm.

Future advances will likely integrate AI-based signal analysis with MIMO architectures, further improving motion compensation and detection accuracy in healthcare, automotive, and other practical domains.

#### 3.1.2. Distributed Radar Sensor

**Figure 3:**
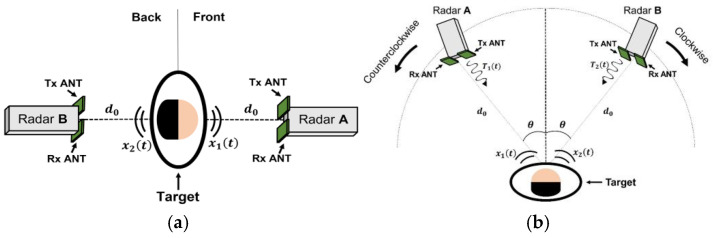
Dual-radar structure for reducing random body movements to obtain accurate vital signs. (**a**) Front-and-rear radar placement; (**b**) configuration considering the asymmetrical movements of human organs [17].

Another promising RBM mitigation strategy involves the use of distributed radar sensors comprising two or more radar systems operating either at the same or different frequencies. This distributed configuration effectively mitigates RBM by capturing radar signals from various perspectives, thereby enhancing the accuracy and reliability of vital sign detection. Figure 3 illustrates the structure of the dual radars arranged at various positions and angles, effectively mitigating RBM to detect accurate vital signals.

- Two 5.8 GHz radars placed at different angles (e.g., 30°, 45°, and 60°) significantly enhanced the SNR, boosting detection accuracy to approximately 97.8% that of single-radar configurations [17].
- A front-and-rear dual-radar setup was tested during treadmill walking with deep learning-based signal restoration (CNN and LSTM), which effectively isolated the respiration signals [18]. This approach maintained high accuracy even under moderate subject motion.
- A 60 GHz FMCW radar that fuses multi-range-bin data for driver monitoring [19] by integrating a Kalman filter with multi-signal fusion achieved a 1.9 bpm lower error than the single-radar approach, reliably detecting breath-hold events.

#### 3.1.3. Multi-View Beamforming Radar

**Figure 4:**
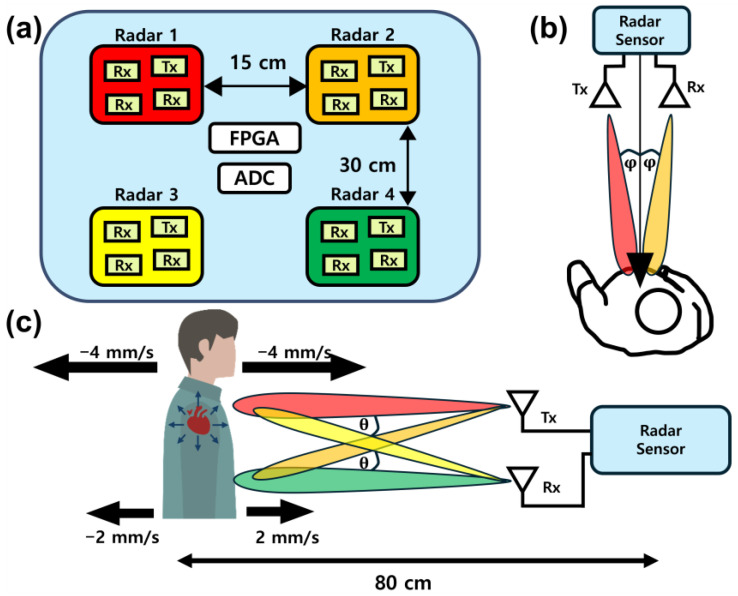
Multi-view beamforming radar system configuration: (**a**) block diagram, (**b**) top view of the simulation setup, and (**c**) 2D side view of the simulation setup (reproduced with permission from the author; Multiview beamforming radar for random body motion cancellation, published by IEEE, 2024) [20].

Multi-view beamforming extends dual-radar ideas by placing multiple radars at different angles to combine measurements (e.g., thoracic vs. abdominal regions) [20]. Figure 4 shows a block diagram of a multi-view beamforming radar system along with views of the simulation setup. Weighted data fusion often uses FFT-based weight selection and reduces RBM artifacts by exploiting different motion patterns in the upper and lower body. Although promising, these methods are sensitive to weight initialization of CNN and have limited large-scale experimental validation.

#### 3.1.4. Single-Chip Single-Antenna Radar

**Figure 5:**
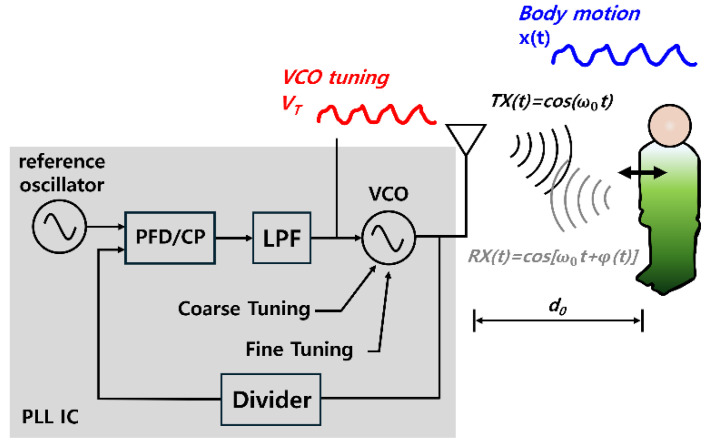
Block diagram of the SIL oscillator radar system with a single-chip single-antenna (reproduced with permission from the author; A single-chip single-antenna radar for remote vital sign monitoring, published by IEEE, 2023) [22].

Compact single-chip solutions using a self-injection-locking (SIL) radar architecture have been explored for portable power-efficient applications [21]. As shown in Figure 5, such systems use a voltage-controlled oscillator (VCO) to transmit and receive signals through a single antenna, thereby reducing hardware complexity and power consumption. Comparative tests on VCO structures based on a combined inductor and capacitor (LCVCO) or ring (RVCO) indicated that the RVCO-based SIL radar offers superior sensitivity, lower power consumption, and a smaller chip footprint. However, challenges remain in detecting weak heartbeat signals in the presence of RBM or high-frequency bands, necessitating further real-world validation.

#### 3.1.5. Multi-Channel Radar Systems Resilient to RBM

**Figure 6:**
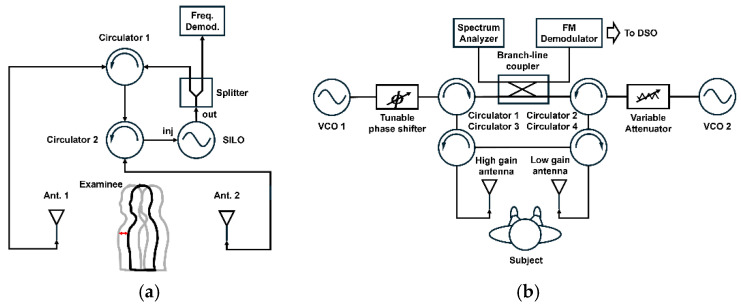
(**a**) Block diagram of the SIL and (**b**) SMIL radar system with multiple antennas (reproduced with permission from the author; Single self-injection-locked radar with two antennas for monitoring vital signs with large body movement cancellation, published by IEEE, 2017. A self- and mutually injection-locked radar system for monitoring vital signs in real time with random body movement cancellation, published by IEEE, 2016) [22,23].

Beyond dual-radar setups, multi-channel radar architectures explicitly target robust performance in RBM [22,23,24,25]. Although CW and FMCW radars are most commonly used for vital sign detection, single-injection-locked (SIL) radars and self/mutually injection-locked (SMIL) systems, depicted in Figure 6, have also been studied. By assigning different gains or weights to each channel, shared motion artifacts can be suppressed while retaining the underlying physiological signals. However, SMIL approaches require careful antenna arrangement and new design trade-offs that must be addressed for widespread adoption.

#### 3.1.6. Accurate Heartbeat Measurement Across Various Body Postures

Advanced algorithms that leverage complex signal models have also been investigated to ensure accurate heartbeat detection in the presence of changing postures and motions [26]. For instance, merging measurements from static and dynamic periods, applying chest-position filtering, and modeling multi-limb composite motion can reduce interference from higher-order harmonics, intermodulation, and DC offsets. The experimental results indicate that such hybrid modeling yields excellent, robust performance for both stationary and moving subjects in real-world conditions.

Overall, innovations in radar architecture, from MIMO to single-chip systems to advanced signal modeling, are converging to address RBM in non-contact vital sign detection. As hardware complexity decreases and signal processing becomes more sophisticated, these systems show increasing potential for practical use in healthcare, automotive monitoring, and smart environments [27,28,29]. Future research should merge machine learning with novel hardware to enhance motion cancellation and deliver highly accurate physiological measurements.

### 3.2. Signal Processing Approaches

#### 3.2.1. Complex Signal Demodulation

**Figure 7:**
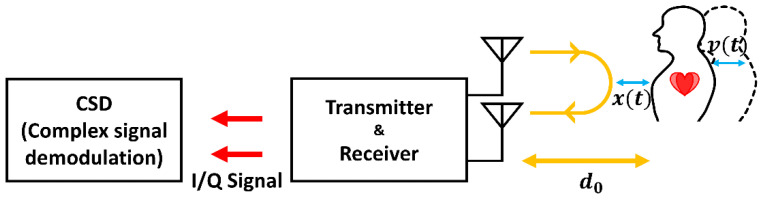
Illustration of the detection setup of the motion modulation effect (reproduced with permission from the corresponding author; Respiration rate measurement under 1-D body motion using single continuous-wave Doppler radar vital sign detection system, published by IEEE, 2016) [31].

Complex signal demodulation preserves the in-phase (I) and quadrature (Q) components of Doppler radar measurements, as shown in Figure 7, allowing better phase recovery despite RBM. Li et al. [30] and Tu et al. [31] demonstrated phase-recovery methods that convert I/Q signals into a complex form to maintain phase continuity. This preserves vital sign information even under moderate motion. Although these methods are straightforward to implement, they require proper DC offset calibration and phase initialization using DC offset estimation, which can limit their applicability in complex or high-speed motion scenarios.

#### 3.2.2. Polynomial Fitting and Adaptive Filter Approaches

**Figure 8:**
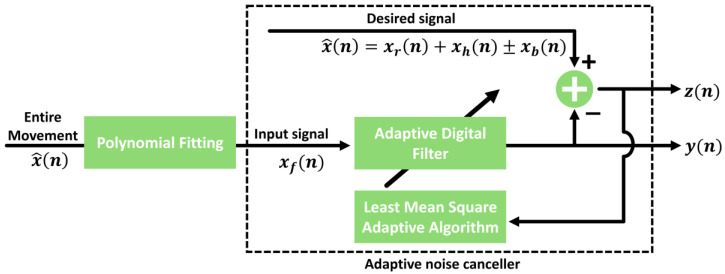
Schematic of adaptive noise cancellation [33].

- Matched filter-assisted polynomial fitting. Lv et al. [32] combined matched filtering with third-order polynomial fitting to remove RBM components at speeds of up to 15 mm/s. Although the matched filter increases the SNR by emphasizing known signal patterns, higher motion speeds (>50 mm/s) still pose challenges because of template mismatch.
- Adaptive noise cancellation (ANC) and N-DCT. As shown in Figure 8, Yang et al. [33] used ANC, polynomial fitting, and N-DCT to enhance signal quality in the presence of RBM by up to 47.6 mm/s. However, an initial ANC learning phase is required, and multi-interference environments can degrade performance.
- Sekak et al. [34] utilized cyclostationary signal processing, which exhibits periodic changes over time, to extract cyclic characteristics in the frequency domain. They introduced several techniques, such as cyclic moments, cyclic autocorrelation, and cyclic cumulants, to differentiate between the first and second order of cycle signals. By analyzing the cyclostationary signal, they obtained vital signal detection with a maximum error of 0.102 Hz for respiration and 0.038 Hz for heartbeat. Measurement results demonstrate that cyclostationary signal analysis can enhance vital sign detection even in the presence of noise and movement, particularly when combined with a bi-static radar system operating at 2.5 GHz.

#### 3.2.3. External Interference Removal Using the Least Squares Method

**Figure 9:**
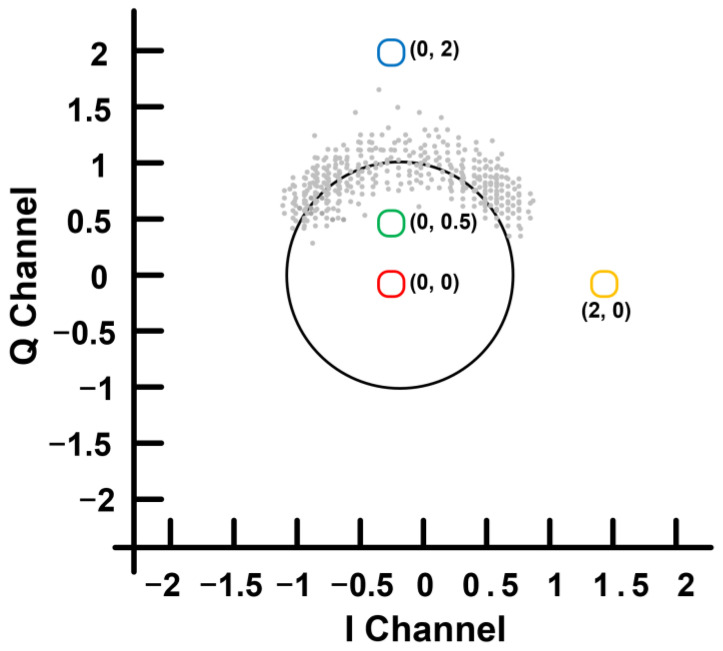
Constellation of simulated signal and four points for DC offset demodulation: (0, 0), (0, 0.5), (0, 2), (2, 0) [35].

Figure 9 shows the least squares method (LSM) with adaptive range bin selection by Dai et al. [35], filtering out unwanted interference by focusing on the most relevant reflection in the range bin. Although LSM is computationally efficient and well suited for real-time processing, multi-target scenarios and strong band-limited interference can limit its effectiveness.

#### 3.2.4. FMCW Radar Integration with Adaptive Filters

**Figure 10:**
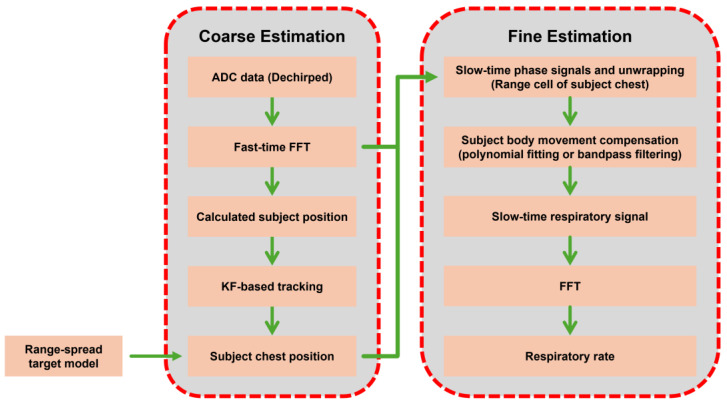
Tracking-aided range-spread (TA-RS) respiration detection method (reproduced with permission from the corresponding author; Tracking-aided respiration detection using radar during large-scale body movements, published by IEEE, 2023) [36].

Ren et al. [36] and Cheng et al. [37] integrated an FMCW radar with adaptive filters to improve detection in the presence of subject movement. Ren et al. [36] employed a Kalman filter-based tracking method and a multi-range bin model, as shown in Figure 10, to account for temporal variations in the signal, enabling the prediction of body movements and the detection of respiration during walking. Adaptive range bin selection, wavelet transforms, and notch filters each contribute to robust motion compensation, with detection accuracies above 94% [37]. However, these approaches can be computationally expensive and sensitive to initial modeling inaccuracies, particularly in multi-target contexts.

#### 3.2.5. Mode Decomposition Techniques

**Figure 11:**
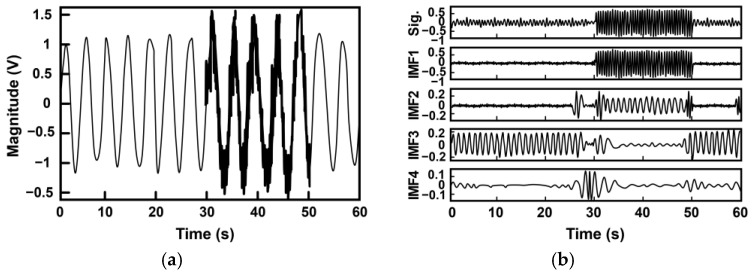
(**a**) Simulated radar sensor signal consisting of a respiration component, heartbeat, and motion artifact. The motion artifact component occupies the segment between t = 30–50 s. (**b**) High-pass filtered sensor output and intrinsic mode functions (the IMFs) within EMD (reproduced by the authors, Cancellation of unwanted Doppler radar sensor motion using empirical mode decomposition; published by IEEE, 2013) [40].

Empirical mode decomposition (EMD), Hilbert vibration decomposition (HVD), and variational mode decomposition (VMD) have been explored for detailed time-frequency analyses of vital signs [38,39,40,41,42]. Figure 11a illustrates a simulated radar sensor signal composed of respiratory and cardiac components, along with motion artifacts [40]. This signal was decomposed into distinct frequency bands using EMD, as presented in Figure 11b.

These mode decomposition techniques are particularly valuable because of their ability to adaptively extract intrinsic mode functions from complex, non-linear, and non-stationary signals. Variants such as improved complete ensemble empirical mode decomposition with adaptive noise (ICEEMDAN), and time-varying filter empirical mode decomposition (TVF-EMD) address mode mixing and intermittence issues [43,44,45].

**Figure 12:**
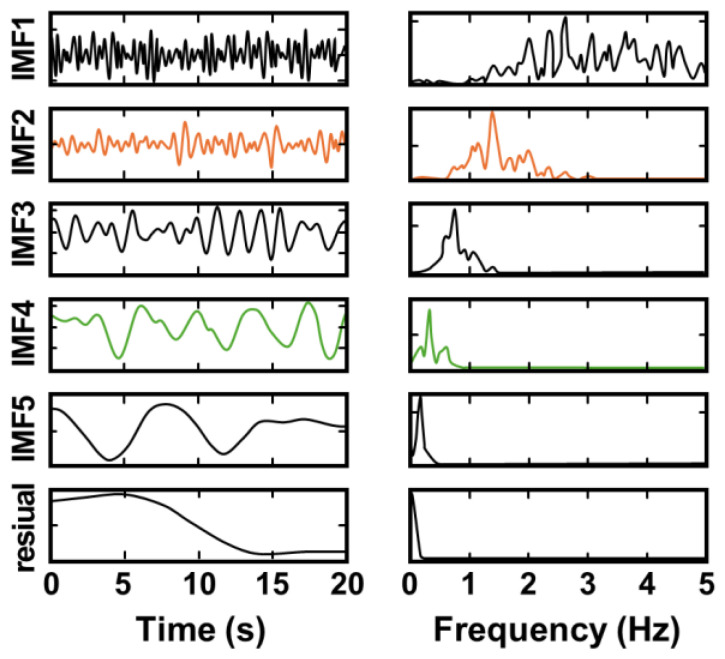
Results of ICEEMDAN decomposition. The left and right sides display the time and frequency domains, respectively (reproduced with permission from the author; A single-chip single-antenna radar for remote vital sign monitoring, published by IEEE, 2023) [43].

- Hu et al. [43] combined ICEEMDAN with the improvised adaptive range bin selection technique to track targets and effectively extract vital signals, even when moving in indoor environments at a walking speed of 1 m/s. The range profile matrix (RPM) and Doppler-range matrix (DRM) were combined to track the movement of the target and dynamically update the position where the signal most strongly reflects the signal, thereby enabling adaptive detection of target location and stable signal detection. Figure 12 shows that the vital information of a moving subject was successfully extracted using the ICEEMDAN method, with significant results that were confirmed through the frequency spectrum.
- Range-Doppler matrix (RDM) of FMCW radar data and a Gaussian interpolation algorithm (GIA) were presented in [44]. RDM is derived from the two-dimensional 2D-FFT of FMCW radar data. A Gaussian interpolation algorithm (GIA) was applied in the Doppler dimension to estimate the target velocity signals. To mitigate large-scale body motion, a robust enhanced trend filtering (RETF) algorithm was employed. To extract respiratory and heartbeat frequencies, the time-varying filter-based empirical mode decomposition (TVF-EMD) algorithm was used. Evaluations with data from seven volunteers using Texas Instrument’s AWR1642 radar achieved accuracies of 93% for respiration and 95% for heart rate detection, effectively handling random body movements without relying on range bin selection, thus avoiding phase wrap issues.
- VMD-based methods improve frequency-band precision. However, optimal parameter tuning (e.g., number of modes, penalty factors) and high computational costs remain barriers to real-time applications. Various studies have explored approaches to optimizing parameter tuning in the VMD method. Qu et al. [46] proposed an improved adaptive parameter variational mode decomposition (IAPVMD) method that integrates the energy loss rate evaluation with the mode discrimination criterion to determine the optimal parameter that would eliminate the RBM. A metaheuristic whale optimization algorithm (WOA)-based parameter tuning method was proposed in [47] to mitigate RBM interference and improve the stability of cardiac component extraction. Variational mode extraction (VME) further refines the VMD by targeting specific frequencies, reducing the complexity of multi-mode decompositions [48,49]. Despite their sophistication, these methods still face challenges in the presence of rapid or unpredictable motions.

### 3.3. Propagation and Environmental Studies

#### 3.3.1. Multi-Radar Data Fusion

**Figure 13:**
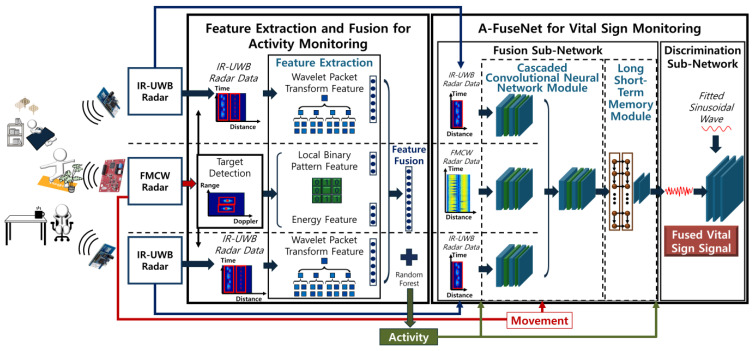
Flow chart of radar data fusion for activity monitoring and vital sign monitoring [50].

To mitigate RBM interference in indoor environments, multi-radar data fusion merges diverse radar modalities, leveraging each sensor’s strengths while minimizing multipath interference, as shown in Figure 13. Yang et al. [50] combined FMCW and impulse radio ultra-wideband (IR-UWB) radars in a linear arrangement at a height of 1.45 m to detect both macro-scale motions (e.g., walking) and micro-scale physiological signals (e.g., heartbeat). Their proposed deep learning framework, A-FuseNet, integrates feature-level fusion and a GAN-based signal optimization step, yielding a 6.9–12.6% improvement in vital sign measurement performance over single-radar baselines. These findings highlight the power of sensor diversity and AI-driven signal processing in enhancing detection accuracy and robustness under complex indoor conditions.

#### 3.3.2. Hybrid Radar Systems for Motion Robust Monitoring

**Figure 14:**
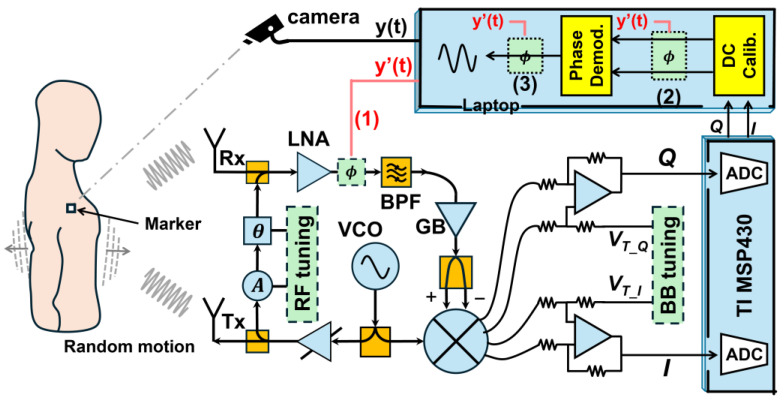
Vital sign monitoring in a dynamic environment via mmWave radar and camera fusion (reproduced with permission from the author; A hybrid radar-camera sensing system with phase compensation for random body movement cancellation in Doppler vital sign detection, published by IEEE, 2013) [51].

Combining FMCW radar with other sensor modalities (e.g., cameras, IR-UWB) provides greater resilience against RBM and environmental interference [51,52]. As shown in Figure 14, the camera measures the RBM, which is then used for phase compensation of the radar signals to enhance the performance of non-contact respiration and heartbeat detection. By dynamically focusing radar beams and applying advanced decomposition methods, vision-assisted radar with variational variance stabilizing mapping (VaR-VSM) detects respiration and heartbeat, even when subjects move their arms or walk. Although the fusion process can introduce computational overhead, it underscores the potential of multi-sensor integration and beamforming to deliver high-accuracy real-time monitoring in dynamic indoor scenarios.

#### 3.3.3. Integrated Neural Networks

**Figure 15:**
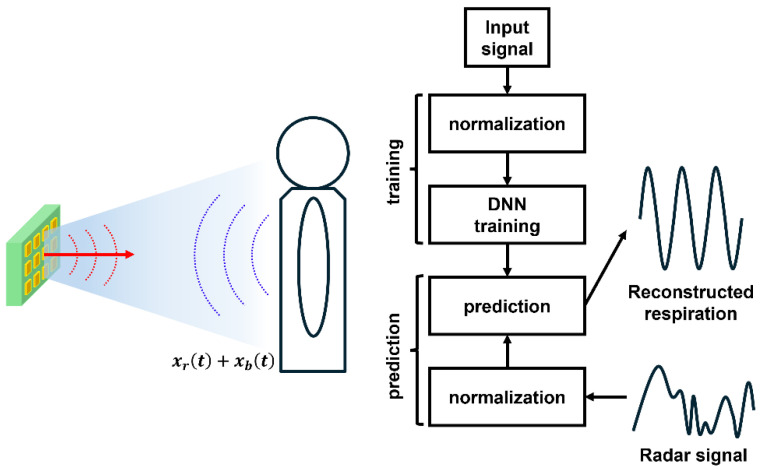
A technique for predicting signal distortion caused by RBM in bio-signal detection by associating nonlinearly extracted signals with previously detected bio-signals using neural network signal processing (reproduced with permission from the author; Deep neural network based body movement cancellation for Doppler radar vital sign detection, published by IEEE, 2019) [54].

Deep learning-based neural networks have become a pivotal method for improving real-time accuracy in radar-based vital sign detection, as shown in Figure 15 [18,53,54,55]. Radar echoes corresponding to respiration and heartbeat feature different frequencies and phases that can easily overlap with RBM noise. Through deep neural networks, researchers can learn the non-linear relationships underlying these signals, enabling the robust separation of RBM components without explicit assumptions about movement patterns. This capability is particularly valuable when conventional signal processing (e.g., convolutional filtering) struggles to disentangle complex mixed signals. The result is stable and accurate real-time detection of respiration and heartbeat, even under moderate to significant subject motion.

A solution was proposed based on leveraging the correlation between body movement intensity and vital signs [55]. Specifically, movement intensity is quantified by analyzing radar signal characteristics, including reflected power, Doppler velocity, and distance. Vital signs are then indirectly estimated through an LSTM-based deep learning model. Additionally, the authors introduced a self-calibration strategy comprising instant calibration and adaptive training, which enables the proposed model to achieve effective generalization across different users without requiring explicit user-specific training data. Experimental results demonstrated robust performance under realistic ambulant conditions, achieving average estimation errors of 5.57 bpm for heart rate and 3.32 bpm for respiration rate, thus significantly enhancing resilience to random body movements.

## 4. Discussion

The field of motion cancellation for CW radar-based vital sign detection continues to evolve. Promising areas for future research include the following:

1. Multi-modal sensor fusion systems

Integrating CW radar with complementary sensor modalities (infrared, ultrasonic, or additional radar types) can enhance robustness against interference and expand coverage [41,43]. Camera–radar fusion and thermal imaging can further boost noise suppression and reduce false positives in multi-subject scenarios.

2. AI-driven signal processing

Deep neural networks (DNNs) and advanced machine learning algorithms can adaptively model the complex interactions between respiratory/cardiac signals and RBM [44,46,47,48,49]. Reinforcement learning can optimize radar parameters in real time, whereas edge computing ensures low-latency deployment. These promising innovations can refine motion cancellation and augment detection accuracy in rapidly changing environments.

3. Low-power and miniaturized radar systems

Research on energy-efficient transceivers, optimized antenna arrays, and small form-factor designs is critical for portable and long-term health monitoring applications. Combining low-power radar hardware with AI-based motion cancellation algorithms can enable continuous real-time operation on mobile or battery-powered devices.

By combining multi-sensor fusion, AI-driven signal processing, and hardware innovations, researchers aim to develop robust, scalable, and energy-efficient CW radar systems capable of accurately measuring respiration and heartbeat even in the presence of random body motion. This convergence of technologies not only addresses current limitations but also opens new prospects for telemedicine, elderly care, and ubiquitous health monitoring.

## 5. Conclusions

Motion artifacts are a critical challenge for CW radar-based vital sign detection systems. Recent advancements in radar architecture, signal processing, and propagation have significantly improved the robustness of RBMs. A variety of methods, such as complex signal demodulation, matched filtering with polynomial fitting, adaptive noise cancellation, least squares filtering, FMCW radar with advanced tracking filters, and mode decomposition techniques, aim to reduce motion artifacts and enhance reliability.

The optimal technique depends on motion severity, computational constraints, and specific system requirements. In many cases, hybrid approaches that combine multiple strategies (e.g., EMD variants with adaptive filtering) produce the most robust results. Ongoing research is striving to achieve real-time performance, multi-target tracking, and greater accuracy under demanding motion conditions. This review examines current techniques and outlines potential future directions for researchers and engineers in the field of non-contact health monitoring using CW radar sensors.

## Data Availability

Data sharing is not applicable.

## Abbreviations

The following abbreviations are used in this manuscript:

ADC	Analog-to-Digital Converter
AI	Artificial Intelligence
ANC	Adaptive Noise Cancellation
CNN	Convolutional Neural Network
CW	Continuous Wave
DNN	Deep Neural Network
DRM	Doppler Range Matrix
EM	Electromagnetic
EMI	Electromagnetic Interference
EMD	Empirical Mode Decomposition
FFT	Fast Fourier Transform
FMCW	Frequency-Modulated CW
GAN	Generative Adversarial Network
HVD	Hilbert Vibration Decomposition
CEEMDAN	Complete Ensemble Empirical Mode Decomposition with Adaptive Noise
ICEEMDAN	Improved Complete Ensemble Empirical Mode Decomposition with Adaptive Noise
IMF	Intrinsic Mode Function
I/Q	In-Phase/Quadrature
IR-UWB	Impulse Radio Ultra-Wideband
LCVCO	LC-Based Voltage-Controlled Oscillator
LFMCW	Linear Frequency-Modulated CW
LNA	Low Noise Amplifier
LO	Local Oscillator
LSM	Least Squares Method
LSTM	Long Short-Term Memory
MIMO	Multi-Input Multi-Output
N-DCT	N-Dimensional Discrete Cosine Transform
PA	Power Amplifier
PMCW	Pulse-Modulated CW
RBM	Random Body Motion
RPM	Range Profile Matrix
RVCO	Ring-Based Voltage-Controlled Oscillator
SIL	Self-Injection Locking
SMIL	Self/Mutually Injection-Locked
SNR	Signal-to-Noise Ratio
TA-RS	Tracking-Aided Range-Spread
TVF-EMD	Time-Varying Filter Empirical Mode Decomposition
VaR-VSM	Vision-Assisted Radar with Variational Variance Stabilizing Mapping
VCO	Voltage-Controlled Oscillator
VMD	Variational Mode Decomposition
VME	Variational Mode Extraction
WMC-VMD	Weighted Multi-Channel Variational Mode Decomposition
